# Early Detection of Thromboembolism during Open Repair for Complex Aortoiliac Aneurysms Using Continuous Noninvasive Lower Limb Oximetry

**DOI:** 10.3400/avd.cr.26-00048

**Published:** 2026-06-27

**Authors:** Naoto Fukunaga, Taiki Takanishi, Tatsuto Wakami, Akio Shimoji, Otohime Mori, Kenta Imai, Tadashi Ikeda, Nobushige Tamura

**Affiliations:** Department of Cardiovascular Surgery, Hyogo Prefectural Amagasaki General Medical Center, Amagasaki, Hyogo, Japan

**Keywords:** thromboembolism, continuous noninvasive lower limb oximetry

## Abstract

A 71-year-old man was referred to our department for open surgery for complex aortoiliac aneurysms. Computed tomography angiography demonstrated aneurysms of the abdominal aorta, bilateral common iliac arteries, and the left internal iliac artery. After stent graft placement in bilateral internal iliac arteries, we planned open surgery. Before cross-clamping the aorta, regional saturation of oxygenation in the left lower limb suddenly decreased from approximately 60% to 30%, despite being stable in the right lower limb. Intraoperative angiography revealed occlusion at the popliteal artery level, for which thromboembolectomy was successfully performed. After that, the aortoiliac aneurysms were replaced. The postoperative course was uneventful.

## Introduction

Open surgery has been a standard of treatment for abdominal aortic aneurysm (AAA), although it is becoming infrequent in the era of endovascular aortic repair.^1)^ During the index surgery, additional procedures, such as renal or lower limb bypass, and thromboembolectomy, result in increased rates of morbidity and mortality.^2)^ Furthermore, thromboembolectomy has been reported as a predictor of postoperative limb and bowel ischemia.^2)^ Nonetheless, it is impossible to detect thromboembolism intraoperatively without causing delay to rescue the limbs.

Continuous noninvasive lower limb oximetry is used to monitor tissue hypoxia in the lower limb and guide the distal perfusion maneuver in patients undergoing femoral arterial cannulation.^3,4)^

Herein, we report the efficacy of continuous noninvasive lower limb oximetry to detect intraoperative thromboembolism during AAA repair.

## Case Report

A 71-year-old man (height: 179 cm, body weight: 58 kg, body mass index: 18.1 kg/m^2^), who had been followed up at a cardiology clinic for a few years, was referred to our department for open surgery for complex aortoiliac aneurysms. He was significant for hypertension, dyslipidemia, lung cancer, paroxysmal atrial fibrillation, and associated cerebral stroke. Despite the event, he was neurologically intact. The lung cancer was a past history and not active at the time of surgery.

He had been on medications for coronary artery disease. Transthoracic echocardiography revealed decreased left ventricular function with moderate mitral regurgitation.

Computed tomography angiography demonstrated an AAA of 54 mm, a right common iliac artery (CIA) of 25 mm, a left CIA of 30 mm, a right internal iliac artery (IIA) of 18 mm, and a left IIA of 28 mm. Atherosclerosis was significant in the aortoiliac lesion.

Because of the complex morphology of the aortoiliac aneurysms, we planned the strategy as follows: the IIA was preserved by reconstructing it first with a stent graft, followed by an anastomosis of a prosthetic graft to the stent graft in a 2-stage manner.^5)^

Therefore, at 2 weeks before the open surgery, we placed the stent grafts (Viabahn stentgraft; W. L. Gore & Associates, Newark, DE, USA) in both the IIAs to exclude the aneurysms under general anesthesia. At the same time, coil embolization of the left inferior gluteal artery was performed to prevent endoleaks. Intraoperative angiography confirmed no evidence of type Ib endoleak at the distal end of the stent grafts.

Computed tomography angiography demonstrated the patency of all the stent grafts and no evidence of type Ib endoleak at the distal end of the stent grafts (**Fig. 1A**).

**Fig. 1:**
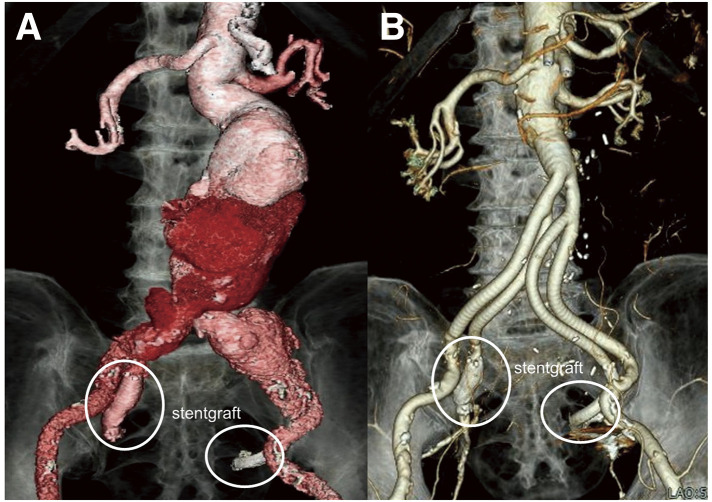
Three-dimensional, post-stent graft replacement (**A**) and postoperative (**B**) computed tomography angiography were depicted. Circles indicated the stent grafts placed into the bilateral internal iliac arteries.

We then performed open surgery for the complex aortoiliac aneurysms. Sensors were placed on the calf of each lower limb to monitor regional saturation of oxygenation (rSO_2_) using near-infrared spectroscopy. The rSO_2_ of both limbs was continuously measured using the INVOS system (Covidien, Boulder, CO, USA). All aneurysms were exposed via laparotomy. Just before cross-clamping the aorta, the rSO_2_ in the left lower limb suddenly decreased from approximately 60% to 30%, despite stable rSO_2_ in the right lower limb (**Fig. 2**). Due to the severe thrombus inside the aneurysms, we considered thromboembolism associated with manipulation of the aneurysms while dissecting the aneurysms. Hence, we proceeded with angiography in the left limb. The left femoral artery was exposed and palpable. Angiography via a 7-Fr sheath placed in the femoral artery revealed an occlusion at the popliteal artery level (**Fig. 3A**, circle). Emergency thromboembolectomy was successfully performed using a 4-Fr Fogarty catheter (Edwards Lifesciences, Irvine, CA, USA) (**Fig. 3B**). The entire procedure of the thromboembolectomy part was performed by cardiologists, and residual thromboemboli were assessed using an intravascular ultrasound. The duration of lower limb ischemia was approximately 3 hrs and 30 min. After thromboembolectomy, the aortoiliac aneurysms were replaced using a quadrifurcated vascular graft (Intergard Knitted Quadrifurcated, size 16 × 8 × 7 mm; Getinge, Gothenburg, Sweden). For the reconstruction of the preserved IIA by placing stent grafts, the distal anastomosis between a branch of the quadrifurcated graft and the stent graft was performed.^5)^

**Fig. 2:**
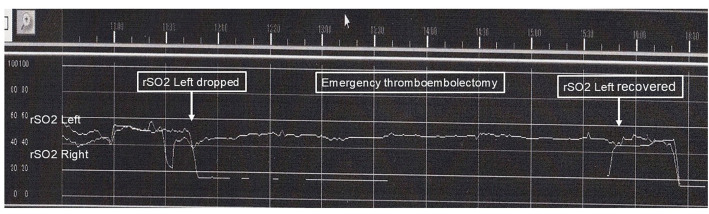
Noninvasive lower limb oximetry was continuously recorded in the left lower limb. Intraoperative events were described in the record. rSO_2_: regional saturation of oxygenation

**Fig. 3:**
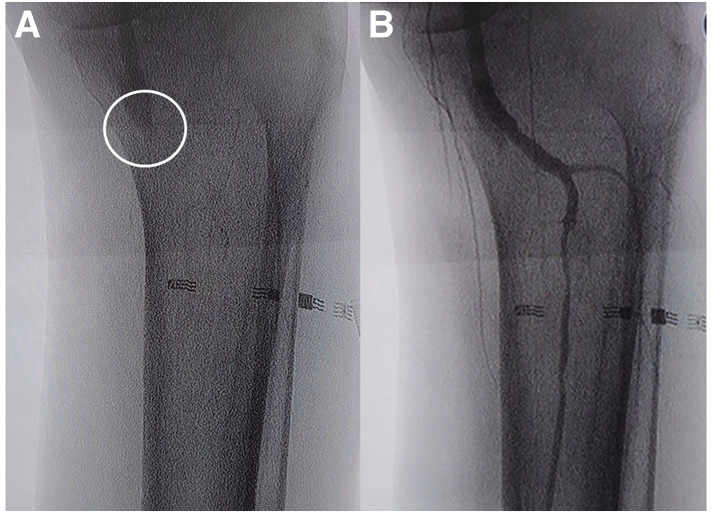
Emergency angiography revealed occlusion of the left popliteal artery (**A**, circle). Revascularization of the popliteal artery was demonstrated after successful intraoperative thromboembolectomy (**B**).

Total operative and aortic cross-clamp duration were 10 hrs 47 min and 31 min, respectively. No transfusion was required. Postoperative computed tomography angiography revealed a patent quadrifurcated graft without pseudoaneurysmal formation at the anastomotic sites (**Fig. 1B**). The peak creatinine kinase level was 5323 U/L on postoperative day 1 (**Fig. 4**). The patient did not complain about any symptoms in the left lower limb. Postoperative ankle–brachial index was 1.13 on the right and 1.17 on the left, compared with preoperative one of 1.13 on the right and 1.18 on the left. The postoperative course was uneventful.

**Fig. 4:**
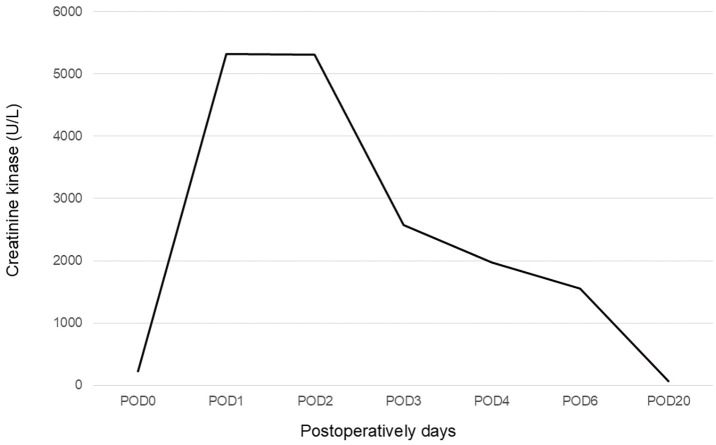
A graph showing postoperative creatinine kinase changes. POD: postoperative day

## Discussion

This case report emphasizes the efficacy of continuous noninvasive lower limb oximetry in diagnosing thromboembolism during open surgery for aortoiliac aneurysms.

Incidence rate of lower limb ischemia due to intraoperative thromboembolism during open surgery was between 4.7% and 7.0%.^6,7)^ The proximal emboli to the iliac, femoral, popliteal, or proximal tibial arteries were less frequently observed compared with emboli to the distal arteries (1.6% vs. 3.1%, respectively). Patients who developed the emboli to the proximal arteries as mentioned above received thromboembolectomy after completion of surgery.^7)^

This means that the clinical diagnosis of acute thromboembolism intraoperatively is challenging, which results in delayed intervention for thromboembolism. Fasciotomy or lower limb amputation would probably be performed.^6)^ Therapeutic fasciotomy was required in approximately 15% of patients who developed compartment syndrome after acute limb ischemia.^8)^ Furthermore, delayed fasciotomy was associated with an increased risk of major amputation at 30 days.^9)^ Thromboembolectomy during AAA repair results in prolonged hospital stay, and increased rates of morbidity and 30-day mortality.^2)^ The previous publications indicated that it was commonly too late when confirming intraoperative thromboembolism.^2,6,8,9)^

In acute arterial occlusion, irreversible changes occur to the nerve in 4–6 hrs, and the muscles in 6–8 hrs. The time allowance to treatment was poor due to the absence of collateral circulation in acute arterial occlusion.^10)^ The present complex case usually takes time to complete the surgery. Therefore, it is essential to detect thromboembolism intraoperatively as soon as it occurred. The patient did not have any complications related to the thromboembolism, due to the prompt thromboembolectomy within the time allowance of tissue damages. Another discussion exists regarding when and where the thrombus came from. It was speculated that thrombus came from the AAA having rich thrombus inside. However, we were not sure that thromboembolism developed while manipulating the aneurysm or after the manipulation. We certainly confirmed that abrupt disappearance of rSO_2_ was recognized before cross-clamping the abdominal aorta.

In this regard, placing the sensors to monitor rSO_2_, which is a simple and noninvasive procedure allows surgeons to promptly detect limb ischemia caused by intraoperative thromboembolism in capacitated patients before interventions are too late. A similar case of revascularization for acute limb ischemia demonstrated the efficacy of rSO_2_ change to assess the tissue perfusion after successful thromboembolectomy.^11)^ In addition, the clinical efficacy of the device applied in peripheral cannulations to detect limb ischemia has been reported.^3,4)^ Based on the efficacy of continuous noninvasive lower limb oximetry for thromboembolism during surgical repair for AAA and similar publications,^3,4,11)^ the present practice reported herein should be applied in cases of open surgery for AAA. In my hospital, the monitoring has been applied routinely in all cases of AAA repair since the present case.

Fat layer thickness decreases the sensitivity when monitoring rSO_2_ in the lower limbs.^12)^ In our case, the patient was not obese, which provided us reliable sensitivity to suspect thromboembolism when rSO_2_ decreased during surgery.

We continue using continuous noninvasive lower limb oximetry during AAA repair to accumulate our experience.

## Conclusion

Continuous noninvasive lower limb oximetry during AAA repair is useful to detect intraoperative thromboembolism.
